# Identification of multiple gene-gene interactions for ordinal phenotypes

**DOI:** 10.1186/1755-8794-6-S2-S9

**Published:** 2013-05-07

**Authors:** Kyunga Kim, Min-Seok Kwon, Sohee Oh, Taesung Park

**Affiliations:** 1 Department of Statistics, Sookmyung Women's University, 100 Cheongpa-ro, Yongsan-gu, Seoul, South Korea; 2 Interdisciplinary Program in Bioinformatics, Seoul National University, 1 Gwanak-ro, Gwanak-gu, Seoul, South Korea; 3 Department of Statistics, Seoul National University, 1 Gwanak-ro, Gwanak-gu, Seoul, South Korea

## Abstract

**Background:**

Multifactor dimensionality reduction (MDR) is a powerful method for analysis of gene-gene interactions and has been successfully applied to many genetic studies of complex diseases. However, the main application of MDR has been limited to binary traits, while traits having ordinal features are commonly observed in many genetic studies (e.g., obesity classification - normal, pre-obese, mild obese and severe obese).

**Methods:**

We propose ordinal MDR (OMDR) to facilitate gene-gene interaction analysis for ordinal traits. As an alternative to balanced accuracy, the use of tau-b, a common ordinal association measure, was suggested to evaluate interactions. Also, we generalized cross-validation consistency (GCVC) to identify multiple best interactions. GCVC can be practically useful for analyzing complex traits, especially in large-scale genetic studies.

**Results and conclusions:**

In simulations, OMDR showed fairly good performance in terms of power, predictability and selection stability and outperformed MDR. For demonstration, we used a real data of body mass index (BMI) and scanned 1~4-way interactions of obesity ordinal and binary traits of BMI via OMDR and MDR, respectively. In real data analysis, more interactions were identified for ordinal trait than binary traits. On average, the commonly identified interactions showed higher predictability for ordinal trait than binary traits. The proposed OMDR and GCVC were implemented in a C/C++ program, executables of which are freely available for Linux, Windows and MacOS upon request for non-commercial research institutions.

## Background

Because most complex biological phenotypes are often affected by multiple genes and environmental factors, the investigation of gene-gene and gene-environment interactions can be essential in understanding the genetic architecture of complex traits [1]. It has been pointed out that focusing only on marginal effects of individual genes may result in low power and a low replication rate in genetic association studies of complex traits [2,3].

Many different methods have been proposed to analyze gene-gene interactions in genetic association studies [4,5], and can be categorized to methods based on regression modeling [6-9], pattern recognition [10,11], and data reduction [12-14]. Recently, machine learning approaches, such as random forest [15], support vector machine [16] and ensemble learning [17], were applied to gene-gene interaction analysis.

While each method has its own advantages and disadvantages, the multifactor dimensionality reduction (MDR) method, a data-reduction approach, is known to have the advantages in examining high-order interactions and detecting interactions without main effects [13,18-20], and has been widely applied to detect gene-gene interactions in many common diseases (see the related literature available on http://epistasis.org). In addition, because the mode of genetic inheritance of a common complex trait is usually unknown a priori, MDR can be more useful to study a complex trait in that it does not require any assumption on genetic model. Since the MDR method was first introduced, it has been extended in many directions. Examples include family data [21], covariate adjustment and quantitative traits [22], the quantitative measure of multi-locus genotype risk [23], and the selection of a parsimonious genetic model [24]. However, the applicability of existing MDR approaches is still restricted mainly to binary traits.

In the MDR analysis for binary traits, multi-locus genotype combinations of a set of genetic variables/markers (e.g., single nucleotide polymorphisms or SNPs) are induced to two levels (e.g., high risk and low risk) of a new binary variable, called an MDR classifier. The induction is conducted via assessing odds of two phenotypic classes for each genotype combination. Among MDR classifiers representing specific marker sets, the single best MDR classifier is selected by evaluating their classification performances, such as cross-validation consistency (CVC). As a result, the corresponding set of genetic markers is identified as having the strongest association with a trait of interest.

While MDR was introduced for binary traits, there is no existing approach that is applicable to ordinal categorical traits. In many genetic association studies, examples of traits having ordinal features are commonly available, such as the obesity classification based on body mass index (e.g., normal, pre-obese, mild obese and severe obese), the diabetes diagnosis based on glucose level (e.g., normal, impaired glucose tolerance and diabetes) and the severity classification of metabolic syndrome. The current application of MDR to these ordinal traits requires to dichotomization of traits by combining several categories, which results in the loss of ordinal information and powers.

In this study, we propose an ordinal MDR (OMDR) approach that enables one to analyze a joint effect of multiple genetic variables on an ordinal categorical trait. The proposed OMDR generates a classifier for each set of genetic markers in the form of a categorical variable with ordinal levels. The performance of each OMDR classifier is evaluated to select the best OMDR classifiers. For performance evaluation, we suggest the use of common ordinal association measures, such as tau-b [25], which test for the trend of directional association between two ordinal variables. By using the ordinal association measures, the performance of OMDR classifier can be evaluated by the degree of tendency of positive association between the observed categories of an ordinal trait and the estimated categories by OMDR.

In addition, we propose a way to report multiple candidates of gene-gene interactions in OMDR as well as MDR analyses. The original MDR approach reports only a single best candidate. This feature can be impractical and/or unreasonable when causal gene-gene interactions are searched for complex traits, especially in a genome-wide scale. Because genome-wide association studies with up to ~1 million SNPs became common, there is a growing need for more efficient criterion to report multiple candidates of gene-gene interactions in the MDR analysis. Thus, we propose a new evaluation measure, generalized cross-validation consistency (GCVC), according to which one can report multiple best gene-gene interactions associated with the ordinal trait. Specifically, a pre-specified number (*K*) of the best classifiers are selected via this GCVC.

Simulations are conducted to investigate performance of the proposed new OMDR method and GCVC. We apply the proposed method to an ordinal obesity trait for body mass index (i.e., normal, pre-obese, mild obese and severe obese) of Age-Related Eye Disease Study data [26].

## Methods

### Overall procedure of OMDR

The OMDR procedure is same as the MDR procedure for binary traits, and consists of multiple steps. First, the dataset is partitioned into *L *(usually equal-size) subsets for *L*-fold cross-validation (CV). For example, *L *= 10 hereafter. Out of 10 subsets, one subset is taken as an independent testing dataset, and the remaining nine subsets are assigned to a training dataset. As a result, a total of 10 CV datasets are generated. Second, all possible OMDR classifiers are constructed for the corresponding combinations of *m *SNPs, and the *K *best ones are selected based on classification performance on a training data for each CV set (see the following two sections for details). Third, the best OMDR classifiers are chosen over all CV sets for the fixed *m*. The predictability of the selected OMDR classifiers is evaluated via the average value of the evaluation measure with a testing dataset over all the 10 CVs. In addition, the selection strength of a particular OMDR classifier is suggested via GCVC*^K ^*which is the number of times the classifier is identified as one of the *K *best classifiers across all the CVs. The best OMDR classifiers across the CVs are chosen if having the maximum predictability and maximum GCVC*^K^*. Finally, the overall best OMDR classifiers are selected based on the predictability and GCVC*^K ^*among the best ones for various values of m, which result from the previous steps. For additional details, refer to the original MDR procedure described in literature [13,27,28].

### OMDR classifier construction

Let 1, 2,..., *J *be classes for an ordinal phenotype of interest. For example, 'low blood pressure (BP)', 'normal' and 'high BP' classes can be viewed as classes 1, 2 and 3, respectively, in the analysis of the BP classification trait. Note that *J *= 2 for a binary trait (e.g., classes1 and 2 respectively for control and case).

Suppose that an *m*-way interaction is under consideration. For the corresponding *m *SNPs, let *n_ij _*be the number of individuals with the *i*th multi-locus genotype and let *n*_+*j *_be the total number of individuals in phenotypic class *j*, where *i *= {1, 2,...,3*^m^*} and *j *= 1, 2,..., *J*. As in MDR, the estimated OR of the class *j *against the class 1 is defined for the *i*th genotype as

(1) $${\hat{\theta }}_{\text{ij}}=\frac{n_{\text{ij}}/n_{i1}}{n_{+j}/n_{+1}}$$

Then the OMDR classifier corresponding to the m given SNPs will assign all individuals with the *i*th multi-locus genotype into the class *c*(*i*) as follows:

(2) $$c\left(i\right)=\underset{j\in \left\{1,\cdots \;,J\right\}}{\text{arg}\text{ max}}{\hat{\theta }}_{\text{ij}}=\underset{j\in \left\{1,\cdots \;,J\right\}}{\text{arg}\text{ max}}\left(\frac{n_{\text{ij}}}{n_{+j}}\right)$$

The final classification results of the OMDR classifier can be described in a *J*×*J *confusion matrix. The (*j*, *k*)th cell of the confusion matrix is denoted as *x_jk _*and indicates the number of individuals in class *j*, who are classified as class *k*:

(3) $$x_{jk}=\underset{\forall i:c\left(i\right)=k}{\sum }n_{\text{ij}}$$

For example, see Table 1 when *J *= 3. Because each of all possible multi-locus genotypes of m given SNPs is represented in a cell of an m-dimensional contingency table, the construction of the corresponding OMDR classifier allows one to reduce the m-dimensional space to one dimensional space. Each constructed OMDR classifier is evaluated by an ordinal association measure, such as tau-b, which assesses concordance between true classes and predicted classes [25].

**Table 1:** Confusion matrix for three-class ordinal phenotype, constructed by an OMDR classifier

Predicted class	True class	1	2	3
1		*x*_11_	*x*_12_	*x*_13_
2		*x*_21_	*x*_22_	*x*_23_
3		*x*_31_	*x*_32_	*x*_33_

### Top-*K *selection and generalized CVC

In order to report multiple causal SNP combinations, we propose the generalized CVC based on top-*K *selection (GCVC*^K^*). After constructing all possible MDR classifiers, each classifier is evaluated respectively with training and testing datasets via a certain evaluation measure of predictability (e.g., tau-b). Then, a pre-specified number (*K*) of the best classifiers having the largest values of the evaluation measure with the training set are selected as top-*K *classifiers for each CV dataset.

Next, the selection results are summarized across all CV datasets in order to suggest multiple best classifiers. The proposed GCVC*^K ^*is defined as below and calculated for each MDR classifier:

(4) $$GCVC^{K}=\overset{L}{\underset{l=1}{\sum }}I_{l}\begin{array}{cc} & where \end{array}\begin{array}{cc} & I_{l}=\left\{\begin{array}{cc} 1 & \text{if the MDR classifier is identified }\\ & \text{as one of top - }K\text{ classifiers at }l^{\text{th}}\text{ CV dataset}\\ 0 & \text{otherwise } \end{array}\right) \end{array}$$

The GCVC*^K ^*indicates how many of the training-test sets support the classifier as the *K *best classifiers in L-fold CV. When *K *= 1, GCVC^1 ^is equal to the original CVC. Note that the proposed GCVC is applicable to both MDR and OMDR.

Via a certain criterion based on GCVC*^K^*, multiple candidates of causal gene-gene interactions with the same order can be reported along with their performance measures (e.g., predictability for training and test datasets). A criterion can be chosen appropriately according to the analysis purpose. We demonstrate possible choices in practice with the following three examples. First, all combinations with GCVC*^K ^* > 0 are reported to search all possible candidates (i.e., exploratory purpose). In other words, every combination that was selected as the K best classifiers at least once during CV will be reported. Second, one can report all combinations with GCVC*^K ^*≥ 9 in 10-fold CV, intending to identify candidates with high selection consistency (i.e., high confidence). This criterion means that these combinations are likely selected with at least 90% chance. Third, 100 plausible candidates are listed up for further studies by reporting top 100 combinations that have the largest values of GCVC*^K^*.

## Results

### Simulation study

**Figure 1:**
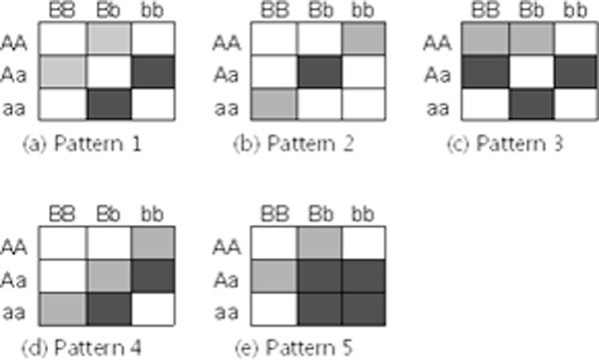
**Simulated patterns of 2-way interactions**. White, light grey and dark grey colors indicate respectively three classes (e.g., normal, low risk, high risk) of an ordinary trait.

An ordinal trait was modeled with 3 classes (e.g., *j *= 1 for normal, *j *= 2 for low risk, *j *= 3 for high risk). Proportion of each class in the population (i.e., *p_j _*= *P*(*j*) 'prevalence' of *j*th class) was set as *p*_1 _= 0.3, *p*_2 _= 0.4 and *p*_3 _= 0.3. A total of 50 SNPs were considered. Among all the SNPs, one pair of SNPs were simulated as a causal factor that has a two-way interaction associated with the ordinal trait; and the remaining SNPs were simulated as non-causal factors. For generating the genotype data of the causal SNPs, five different interaction patterns were developed for the ordinal trait (Figure 1). While fixing minor allele frequencies (0.3 and 0.5), prevalences and interaction pattern, we simulated 3 different sets of ORs of each class for each multi-locus genotype in order to vary the strength of genetic effects. Based on given ORs, probabilities of each class for each multi-locus genotype (i.e., *p*_*j*|*i *_= *P*(*j *| the *i*th genotype) 'penetrance' of *j*th class for *i*th genotype) were computed under the Hardy-Weinberg equilibrium assumption for each SNP. As a result, 15 different genetic models were developed (Table S1 in Additional file 1). For each genetic model, 100 replicated datasets were generated. Each simulated dataset consists of 1000 samples. For comparison, we further generated a binary trait by assigning the first two classes of the simulated ordinary trait (i.e., normal and low risk) to 'control' and the third class (i.e., high risk) to 'case'. Thus the prevalence of case is expected to be 0.3 for the binary trait. The proposed OMDR and the original MDR were applied to the simulated datasets. The 10-fold CV and tau-b were employed to assess the performance of classifiers. All possible single-, two- and three-locus classifiers were evaluated. Different choices of *K *= 1, 2, 3 were considered to examine the effect of choice on GCVC*^K^*.

The performances of the new OMDR method were investigated in terms of power, fitness, predictability, and selection stability. The empirical power was defined as the proportion of 100 replicated datasets in which the true causal SNP combination was detected as the best classifier. The fitness and predictability were measured respectively via average training tau-b (TRTB) and testing tau-b (TSTB) values across 100 replicated datasets. Average GCVC was used to assess selection stability. First, we observed fairly good empirical power across various genetic models (Table 2). Especially two-locus OMDR classifiers show high empirical power of about 90% on average, and 100% for two third of genetic models. Second, the overall predictability of two-locus classifiers (average TSTB = 0.285) was slightly higher than or similar to three-locus classifiers (average TSTB = 0.276) while single-locus classifiers had relatively low predictability (average TSTB = 0.139). Therefore, the proposed OMDR tends not to choose lower-order interactions than the order of the true causal interaction. Third, two-locus classifiers were most stably selected (average GCVC = 92.0%) compared to others, especially three-locus classifiers (average GCVC = 48.3%). Thus the OMDR selected two-locus classifiers as a final best model more likely than three-locus ones. This indicates that the OMDR would choose true causal interactions while avoiding over-fitting. As expected, higher-order classifiers showed higher fitness (average TRTB = 0.162, 0.304 and 0.336, respectively, for single-, two-, and three-locus classifiers), and that the difference in average TRTB between two- and three-locus classifiers was not great.

**Table 2:** Performance of OMDR

	**Single-locus classifier**				**Two-locus classifier**				**Three-locus classifier**			
			
	**EP (SEP)**	**GCVC**	**TSTB**	**TRTB**	**EP**	**GCVC**	**TSTB**	**TRTB**	**EP (TEP)**	**GCVC**	**TSTB**	**TRTB**
		
Model 11	0.38 (0.25)	0.689	0.055	0.097	1.00	1.000	0.444	0.443	1.00 (0.05)	0.514	0.429	0.455
Model 12	0.27 (0.14)	0.667	0.043	0.093	1.00	1.000	0.264	0.278	1.00 (0.05)	0.518	0.256	0.315
Model 13	0.26 (0.16)	0.615	0.046	0.095	0.93	0.883	0.139	0.174	0.87 (0.05)	0.458	0.128	0.224
Model 21	1.00 (0.55)	0.883	0.159	0.175	1.00	1.000	0.395	0.401	1.00 (0.04)	0.480	0.388	0.417
Model 22	0.99 (0.53)	0.881	0.150	0.167	1.00	1.000	0.297	0.306	1.00 (0.05)	0.432	0.267	0.330
Model 23	0.95 (0.54)	0.874	0.115	0.134	0.99	0.993	0.230	0.249	1.00 (0.08)	0.504	0.224	0.292
Model 31	1.00 (1.00)	1.000	0.225	0.229	1.00	1.000	0.514	0.513	1.00 (0.03)	0.507	0.505	0.527
Model 32	1.00 (1.00)	0.997	0.192	0.197	1.00	1.000	0.373	0.375	1.00 (0.06)	0.531	0.356	0.397
Model 33	0.44 (0.31)	0.714	0.061	0.099	1.00	0.997	0.194	0.208	0.99 (0.05)	0.537	0.185	0.245
Model 41	1.00 (0.56)	0.904	0.131	0.144	1.00	0.999	0.252	0.264	1.00 (0.06)	0.544	0.255	0.300
Model 42	1.00 (0.55)	0.887	0.149	0.164	0.90	0.883	0.204	0.217	0.94 (0.05)	0.454	0.189	0.247
Model 43	0.73 (0.41)	0.816	0.083	0.107	0.28	0.485	0.068	0.146	0.23 (0.02)	0.346	0.061	0.197
Model 51	1.00 (0.51)	0.865	0.341	0.355	1.00	1.000	0.478	0.484	1.00 (0.05)	0.480	0.477	0.512
Model 52	1.00 (0.51)	0.885	0.247	0.263	1.00	0.998	0.334	0.344	1.00 (0.05)	0.473	0.325	0.377
Model 53	0.91 (0.46)	0.860	0.092	0.117	0.38	0.557	0.086	0.153	0.47 (0.04)	0.426	0.099	0.204
		
Average	0.80 (0.50)	0.836	0.139	0.162	0.90	0.920	0.285	0.304	0.90 (0.05)	0.483	0.276	0.336

It is presented with EP (empirical power), average GCVC (*K *= 1), average TSTB (testing tau-b), average TRTB (training tau-b), and their average values over models. Note that true causal factor was a two-locus classifier (i.e., two-way interaction). SEP indicates EP of single-locus classifier whose EP is largest among all single-locus classifiers included in the true causal interaction. TEP indicates EP of three-locus classifier whose EP is largest among all three-locus classifiers containing the true causal interaction.

**Figure 2:**
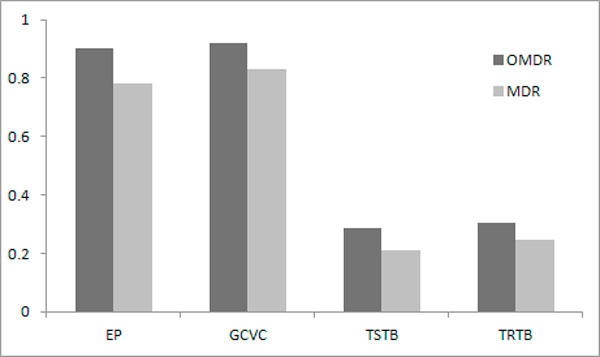
**Comparison between OMDR and MDR**. Performance of OMDR and MDR is compared via EP (empirical power), average GCVC (*K *= 1), average TSTB (testing tau-b), average TRTB (training tau-b), and their average values over models. Note that true causal factor was a two-locus classifier (i.e., two-way interaction), and all two-locus classifiers were searched by both methods.

We compared the performance between the OMDR method and the original MDR method. Overall, the OMDR showed better performance than the MDR across all performance measures (Figure 2). Especially, we observed higher empirical power and better selection stability for the OMDR than for the MDR. Also, predictability and fitness indicated that the OMDR (on average, TSTB = 0.285, TRTB = 0.304) outperformed the MDR (on average, TSTB = 0.209, TRTB = 0.247) across all genetic models.

The effect of *K *on the OMDR was examined with different *K *= 1, 2 and 3. Because we simulated with a single causal two-way interaction, selected classifiers must include false positives when *K *= 2 or 3 were chosen for top-*K *selection. In most genetic models, the causal interaction was identified as the best classifiers (i.e., true positives). Thus the second best or the third best classifiers were falsely identified (i.e., false positives). We compared the selection stability and predictability between true and false positives. While true positives were selected with high stability (average GCVC = 92.0~94.7%), false positives were selected with very low stability (average GCVC = 3.4~43.5%). These limited results imply that one can avoid false positives, which the OMDR produces with large *K*, by further screening out the selected classifiers with low GCVC. Thus incorrect choices of *K *would not fail the OMDR although further investigation on the choice of *K *is required. Note that predictability was higher for true positives (average TSTB = 0.285~0.288) that for false positives (average TSTB = 0.106~0.133).

### Analysis of AREDS body-mass index data

In order to demonstrate the proposed OMDR, we applied it to a body-mass index (BMI) category phenotype from the National Eye Institute Age-Related Eye Disease Study (AREDS). While the AREDS was originally designed to investigate the clinical course of age-related macular degeneration (AMD), the data contains other information on medical history, clinical status, life condition, and physical measurements, including BMI. A total of 313 subjects with and 149 without AMD were genotyped on Affymetrix 100K genotyping platform. The detailed information on this data is available in Maller et al. [26]. Prior to the analysis, we conducted a pre-process on a total of 109,924 SNPs by excluding SNPs whose total genotyping rate < 99.5%, minor allele frequency < 0.05, or p-value from Hardy-Weinberg equilibrium test ≤ 10^-3^. As a result, a total of 87,260 SNPs remained for the analysis.

According to the international BMI classification [29], an adult person can be classified as normal when 18.5 ≤ BMI < 25, and as overweight when BMI ≥ 25. The overweight class is further divided into pre-obese, obese class I, obese class II and obese class III (Table 3). Using this classification, we defined a four-class ordinal phenotype 'OD' (i.e., normal, pre-obese, mild obese and severe obese) to identify genes and gene-gene interactions associated with obesity via the proposed OMDR. The sample sizes are 141, 194, 87 and 38 for normal, pre-obese, mild obese and severe obese classes, respectively. In addition, two binary phenotypes 'B1' and 'B2' (i.e., normal and overweight; non-obese and obese) were defined and analyzed via the current MDR for dichotomous phenotypes for the comparison purpose.

**Table 3:** Obesity phenotypes based on BMI classification

WHO classification		Ordinary Category OD	Binary Category	
			
			B1	B2
Normal	18.5 ≤ BMI < 25	Normal	Normal	Non-obese

Pre-obese	25 ≤ BMI < 30	Pre-obese	Overweight	

Obese class I	30 ≤ BMI < 35	Mild obese		Obese

Obese class II	35 ≤ BMI < 40	Severe obese		

Obese class III	BMI ≥ 40			

For the ordinal phenotype (OD) and two binary phenotypes (B1 and B2), the proposed OMDR and the current MDR were respectively applied to identify SNPs associated with obesity. We used *K *= 300 to select multiple best MDR classifiers for each of 1~4-way interactions. The 10-fold CV and tau-b were employed to assess the performance of classifiers. All 87,260 SNPs were first searched for one-way effects on obesity. Then, to reduce the computational burden, we examined all pairwise combinations of the top-300 SNPs with main effects. Similarly, three- and four-way combinations were searched only for the SNPs that were selected with top-300 two- and three-way interactions, respectively.

For the top-300 SNPs identified with main effects, the average GCVC was 6.67 for OD while it was 5.98 and 5.96, respectively for B1 and B2. We also observed that more SNPs were identified with high GCVC for OD than B1 and B2. For example, the number of SNPs showing GCVC = 10 is 58, 22 and 26 respectively for OD, B1 and B2. The number of SNPs with GCVC ≥ 9 for OD is also about twice the number of those for B1 and B2. These patterns are stronger for 2~4-way interactions (Figure S1 in Additional file 2). While the binary MDR method identified most interactions with low GCVC, the OMDR approach detected a higher number of interactions with high CVC. Among top-300 two-way interactions, 111 have GCVC of 10 for OD while 7 and 10 do for B1 and B2, respectively. Similarly, 92 three-way and 49 four-way interactions show GCVC of 10 for OD while only a few do for the binary phenotypes. These results indicate that, with a high level of selection consistency, the proposed OMDR would detect more interactions than the original MDR for binary phenotypes.

While no SNP was selected with main effect across all three phenotypes, two SNPs were commonly identified by OD and B1. Fourteen SNPs identified for OD were also selected for B2. For these commonly selected SNPs, we investigated average tau-b values on training and test datasets as well as GCVC (Table 4). All of these SNPs show better performance both on model fitting and prediction for the ordinal phenotype (average tau-b = 0.365 and 0.317 for training and testing datasets on average across the commonly selected SNPs) than for the binary phenotypes (average tau-b = 0.175 and 0.081 for training and testing datasets on average across the commonly selected SNPs). Furthermore, we observed higher GCVC for OD than for B1 and B2. In other words, the SNP selection was more strongly supported by 10-fold CV for the ordinal phenotype than for the binary phenotypes. These results would imply that the proposed OMDR provides more consistent results than the original MDR for binary phenotypes.

**Table 4:** Commonly identified SNPs with main effects on obesity.

SNP	OD			B1/B2		
		
	GCVC	Average tau-b		GCVC	Average tau-b	
				
		Train	Test		Train	Test
rs1975743*	9	0.371	0.352	9	0.184	0.144
rs10504852*	9	0.357	0.345	7	0.165	0.109
rs3856570	10	0.402	0.397	10	0.237	0.233
rs166315	10	0.369	0.367	5	0.169	0.035
rs997682	10	0.369	0.363	6	0.192	0.246
rs1980774	10	0.367	0.361	6	0.192	0.245
rs354935	9	0.387	0.376	9	0.175	0.155
rs10515827	9	0.360	0.341	4	0.162	0.061
rs2006709	8	0.361	0.330	7	0.166	0.100
rs959175	7	0.367	0.319	4	0.158	-0.066
rs2000862	7	0.359	0.289	5	0.166	0.008
rs4780469	7	0.353	0.284	5	0.165	0.018
rs1009829	5	0.361	0.298	4	0.169	-0.002
rs4779937	5	0.355	0.261	4	0.166	0.029
rs9297682	4	0.358	0.205	5	0.164	0.019
rs10508706	4	0.353	0.192	6	0.166	0.071

SNPs with * were identified for OD and B1; SNPs with no * were identified for OD and B2.

In order to examine the biological significance, we further investigated whether the top-300 SNPs with main effects are mapped to one of the known obesity-related genes that were represented on Affymetrix 100K genotyping platform. For each phenotype, only three SNPs were identified in the known obesity-related genes. However, those obesity-related SNPs were identified more consistently by the OMDR (average GCVC = 7.33 for OD) than by the original MDR (average GCVC = 5 and 6.33 for B1 and B2, respectively). Note that the famous obesity-associated gene *FTO *was detected only via OMDR. Also, we found that the *ARL6 *gene was detected with larger tau-b for OD than for B2 (see rs3856570 in Table 4).

In addition, various values of *K *(*K *= 1, 2,..., 1000) were further used to search for possible causal SNPs with main effects via the OMDR and the original MDR methods. As we increased *K *(i.e., considered to select a larger number of possible causal SNPs), we identified more obesity-related SNPs using the OMDR approach than using the current MDR, and the gap seems increasing. For example, with *K *= 1000, we identified four more SNPs in known obesity-related genes for OD than for B1 and B2.

## Conclusions and discussion

In this paper, we developed the OMDR approach that facilitates the MDR analysis for an ordinal phenotype. The construction process for OMDR classifiers is a straightforward extension of the process for the existing MDR classifiers. For selecting good classifiers, the performance of the OMDR classifiers has to be evaluated via an evaluation measures. We proposed the use of an ordinal association measure, specifically tau-b, for some reasons. First, tau-b along with likelihood ratio and normalized mutual information has been known to outperform other evaluation measures in MDR, including balanced accuracy, misclassification error, specificity and sensitivity [28,30]. Second, tau-b and other ordinal association measures would be natural choices to assess the association between the true and the predicted classes (see Table 1), both of which are ordinal, in that they utilize the information on positive trend in classification results. In addition, tau-b can be readily employed to OMDR without modification.

While designed for the analysis of genuine ordinal categorical traits, the OMDR method can also be used to analyze a continuous trait by approximating it as an ordinary category trait. Currently, the MDR analysis for a continuous trait is conducted mostly by binarizing it with a certain cut-off. Compared to binary approximation, the ordinary approximation can be more powerful because it preserves more information on the continuous trait. The empirical study with a real data demonstrated that the OMDR approach would produce more consistent results and be more powerful than the original MDR approach for binary traits, in terms of GCVC and the number of the classifiers identified with high selection consistency, respectively.

Nowadays, the genome-wide association studies with the genotype data produces up to ~1 million SNPs. Reporting one single best candidate is impractical and/or unreasonable when causal gene-gene interactions are searched for complex traits in a genome-wide scale. Thus, we proposed GCVC with the top-*K *selection to report multiple candidates of gene-gene interactions in OMDR as well as MDR analyses. When one searches for few but possibly strong candidates for gene-gene interactions, a small value of *K *would be appropriate. On the other hand, a large value of *K *can be used for detecting many candidates including ones with mild effects on traits. Note that the choice of *K *would be critical for both power increase and false-positive control, which requires a further investigation. However, our simulations suggested that false positives can be screened out with very low GCVC values and low predictability values. We also investigated the null distribution of GCVC*^K ^*with different *K *= 1, 2, 3, based on a small simulation, and found that the choice of *K *did not dramatically affect the null distribution of GCVC*^K ^*(data not shown).

The original MDR for binary traits (e.g., disease status) compares the estimated ORs between two classes (e.g., case vs. control), and determines the class with larger estimated OR as the predicted class. When the estimated ORs are same for both classes, one class is usually specified for the prediction purpose (e.g., high risk). Similarly, more than one class can happen to have the same maximum value of the estimated OR (i.e., a tie in the estimated OR among classes; multiple values of *c*(*i*)) in the OMDR approach. There might be many possible options to address this tie problem. For examples, the class with the smallest or largest *K *can be used as the predicted class among the tied classes. In our analysis, we chose the largest class for prediction in tied cases following the original MDR approach.

## Competing interests

The authors declare that they have no competing interests.

## Authors' contributions

KK, MSK and TSP designed the study and developed the methodologies. KK and MSK planned and performed the simulation analysis. SO completed the real data analysis. KK and TSP wrote the manuscript. All authors read and approved the final manuscript.

## Supplementary Material

Additional file 1 **Simulation settings based on 15 genetic models**. OR_*j*1 _is presented as odd ratio of class *j *against class 1 for each two-locus genotype along with the corresponding penetrance (*p*_*j*|*i *_in parentheses. Minor allele frequencies (MAFs) of 0.5 are used in models with patterns 1 and 2; MAFs of 0.3 are used in models with other patterns.) Click here for file

Additional file 2 **GCVC value distribution of top-300 OMDR classifiers for 2~4-way interactions from real data analysis**. Click here for file

## Declarations

The publication costs for this article were funded by the corresponding author.

This article has been published as part of *BMC Medical Genomics *Volume 6 Supplement 2, 2013: Selected articles from the Second Annual Translational Bioinformatics Conference (TBC 2012). The full contents of the supplement are available online at http://www.biomedcentral.com/bmcmedgenomics/supplements/6/S2.

## References

[B1] Moore JH The ubiquitous nature of epistasis in determining susceptibility to common human diseases Hum Hered 2003 56 73 82 10.1159/000073735 14614241

[B2] Culverhouse R Suarez BK Lin J Reich T A perspective on epistasis: limits of models displaying no main effect Am J Hum Genet 2002 70 461 471 10.1086/338759 11791213 PMC384920

[B3] Marchini J Donnelly P Cardon LR Genome-wide strategies for detecting multiple loci that influence complex diseases Nat Genet 2005 37 413 417 10.1038/ng1537 15793588

[B4] Musani SK Detection of gene x gene interactions in genome-wide association studies of human population data Hum Hered 2007 63 67 84 10.1159/000099179 17283436

[B5] Cordell HJ Detecting gene-gene interactions that underlie human diseases Nat Rev Genet 2009 10 392 404 19434077 10.1038/nrg2579 PMC2872761

[B6] Cordell HJ Epistasis: what it means, what it doesn't mean, and statistical methods to detect it in humans Hum Mol Genet 2002 11 2463 2468 10.1093/hmg/11.20.2463 12351582

[B7] Kooperberg C Ruczinski I Identifying interacting SNPs using Monte Carlo logic regression Genet Epidemiol 2005 28 157 170 10.1002/gepi.20042 15532037

[B8] Millstein J Conti DV Gilliland FD Gauderman WJ A testing framework for identifying susceptibility genes in the presence of epistasis Am J Hum Genet 2006 78 15 27 10.1086/498850 16385446 PMC1380213

[B9] Park MY Hastie T Penalized logistic regression for detecting gene interactions Biostatistics 2008 9 30 50 17429103 10.1093/biostatistics/kxm010

[B10] Motsinger-Reif AA Dudek SM Hahn LW Ritchie MD Comparison of approaches for machine-learning optimization of neural networks for detecting gene-gene interactions in genetic epidemiology Genet Epidemiol 2008 32 325 340 10.1002/gepi.20307 18265411

[B11] Sherriff A Ott J Applications of neural networks for gene finding Adv in Genet 2001 42 287 297 11037328 10.1016/s0065-2660(01)42029-3

[B12] Nelson MR A combinatorial partitioning method to identify multilocus genotypic partitions that predict quantitative trait variation Genome Res 2001 11 458 470 10.1101/gr.172901 11230170 PMC311041

[B13] Ritchie MD Hahn LW Roodi N Bailey LR Dupont WD Parl FF Moore JH Multifactor-dimensionality reduction reveals high-order interactions among estrogen-metabolism genes in sporadic breast cancer Am J Hum Genet 2001 69 138 147 10.1086/321276 11404819 PMC1226028

[B14] Zhang H Bonney G Use of classification trees for association studies Genet Epidemiol 2000 19 323 332 10.1002/1098-2272(200012)19:4<323::AID-GEPI4>3.0.CO;2-5 11108642

[B15] Bureau A Identifying SNPs predictive of phenotype using random forests Genet Epidemiol 2005 28 171 182 10.1002/gepi.20041 15593090

[B16] Chen S A support vector machine approach for detecting gene-gene interaction Genet Epidemiol 2008 32 152 167 10.1002/gepi.20272 17968988

[B17] Zhang Z An ensemble learning approach jointly modelling main and interaction effects in genetic association studies Genet Epidemiol 2008 32 285 300 10.1002/gepi.20304 18205210 PMC3572743

[B18] Hahn LW Ritchie MD Moore JH Multifactor dimensionality reduction software for detecting gene-gene and gene-environment interactions Bioinformatics 2003 19 376 382 10.1093/bioinformatics/btf869 12584123

[B19] Moore JH Gilbert JC Tsai CT Chiang FT Holden T Barney N White BC A flexible computational framework for detecting, characterizing, and interpreting statistical patterns of epistasis in genetic studies of human disease susceptibility J Theor Biol 2006 241 252 261 10.1016/j.jtbi.2005.11.036 16457852

[B20] Ritchie MD Hahn LW Moore JH Power of multifactor dimensionality reduction for detecting gene-gene interactions in the presence of genotyping error, missing data, phenocopy, and genetic heterogeneity Genet Epidemiol 2003 24 150 157 10.1002/gepi.10218 12548676

[B21] Martin ER Ritchie MD Hahn L Kang S Moore JH A novel method to identify gene-gene effects in nuclear families: the MDR-PDT Genet Epidemiol 2006 30 111 123 10.1002/gepi.20128 16374833

[B22] Lou XY Chen GB Yan L Ma JZ Zhu J Elston RC Li MD A generalized combinatorial approach for detecting gene-by-gene and gene-by-environment interactions with application to nicotine dependence Am J Hum Genet 2007 80 1125 1137 10.1086/518312 17503330 PMC1867100

[B23] Chung Y Lee SY Elston RC Park T Odds ratio based multifactor-dimensionality reduction method for detecting gene-gene interactions Bioinformatics 2007 23 71 76 10.1093/bioinformatics/btl557 17092990

[B24] Lee SY Chung Y Elston RC Kim Y Park T Log-linear model-based multifactor dimensionality reduction method to detect gene-gene interactions Bioinformatics 2007 23 2589 2595 10.1093/bioinformatics/btm396 17872915

[B25] Agresti A Categorical Data Analysis 2002 Wiley-Interscience

[B26] Maller JB Fargeness JA Reynolds RC Neale BM Daly MJ Seddon JM Variation in complement factor 3 is associated with risk of age-related macular degeneration Nat Genet 2007 39 1200 1201 10.1038/ng2131 17767156

[B27] Moore JH Williams SM New strategies for identifying gene-gene interactions in hypertension Ann Med 2002 34 88 95 10.1080/07853890252953473 12108579

[B28] Namkung J Kim K New evaluation measures for multifactor dimensionality reduction classifiers in gene-gene interaction analysis Bioinformatics 2009 25 338 345 10.1093/bioinformatics/btn629 19164302

[B29] WHO Obesity: preventing and managing the global epidemic, Report of a WHO Consultation World Health Organization Technical Report 2000 Series 894 11234459

[B30] Bush WS Alternative contingency table measures improve the power and detection of multifactor dimensionality reduction BMC Bioinformatics 2008 9 238 244 10.1186/1471-2105-9-238 18485205 PMC2412877

