# Supplementary material for: Identification of multiple gene-gene interactions for ordinal phenotypes
Source: BMC Med Genomics. 2013 May 7;6(Suppl 2):S9. doi: 10.1186/1755-8794-6-S2-S9 (PMC3654913; doi:10.1186/1755-8794-6-S2-S9)
Supplement: Additional file 1 — Simulation settings based on 15 genetic models. ORj1 is presented as odd ratio of class j against class 1 for each two-locus genotype along with the corresponding penetrance (pj|i in parentheses. Minor allele frequencies (MAFs) of 0.5 are used in models with patterns 1 and 2; MAFs of 0.3 are used in models with other patterns.) [file 1755-8794-6-S2-S9-S1.pdf]

**Table S1.** Simulation settings based on 15 genetic models. $OR_{j1}$ is presented as odd ratio of class $j$ against class 1 for each two-locus genotype along with the corresponding penetrance ($p_{j|i}$) in parentheses. Minor allele frequencies of 0.5 are used in models with patterns 1 and 2; MAFs of 0.3 are used in models with other patterns.

| Pattern 1 | Model 11 | | Model 12 | | Model 13 | |
|---|---|---|---|---|---|---|
| Genotype | $OR_{21}$ ($p_{2\|i}$) | $OR_{31}$ ($p_{3\|i}$) | $OR_{21}$ ($p_{2\|i}$) | $OR_{31}$ ($p_{3\|i}$) | $OR_{21}$ ($p_{2\|i}$) | $OR_{31}$ ($p_{3\|i}$) |
| AABB | 0.33 (0.233) | 0.452 (0.239) | 0.32 (0.178) | 0.995 (0.410) | 0.33 (0.202) | 0.72 (0.334) |
| AABb | 10 (0.690) | 5 (0.259) | 3.04 (0.575) | 2 (0.284) | 2 (0.548) | 1.2 (0.247) |
| AAbb | 0.4 (0.291) | 0.3 (0.164) | 0.61 (0.371) | 0.38 (0.173) | 0.8 (0.400) | 0.6 (0.225) |
| AaBB | 10 (0.690) | 5 (0.259) | 3.04 (0.575) | 2 (0.284) | 2 (0.548) | 1.2 (0.247) |
| AaBb | 0.4 (0.291) | 0.3 (0.164) | 0.61 (0.371) | 0.38 (0.173) | 0.8 (0.400) | 0.6 (0.225) |
| Aabb | 5 (0.377) | 10 (0.566) | 2 (0.398) | 3.04 (0.453) | 1.2 (0.348) | 2 (0.435) |
| aaBB | 0.3 (0.222) | 0.4 (0.222) | 0.38 (0.239) | 0.61 (0.288) | 0.6 (0.308) | 0.8 (0.308) |
| aaBb | 5 (0.377) | 10 (0.566) | 2 (0.398) | 3.04 (0.453) | 1.2 (0.348) | 2 (0.435) |
| aabb | 0.3 (0.222) | 0.4 (0.222) | 0.38 (0.239) | 0.61 (0.288) | 0.6 (0.308) | 0.8 (0.308) |

| Pattern 2 | Model 21 | | Model 22 | | Model 23 | |
|---|---|---|---|---|---|---|
| Genotype | $OR_{21}$ ($p_{2\|i}$) | $OR_{31}$ ($p_{3\|i}$) | $OR_{21}$ ($p_{2\|i}$) | $OR_{31}$ ($p_{3\|i}$) | $OR_{21}$ ($p_{2\|i}$) | $OR_{31}$ ($p_{3\|i}$) |
| AABB | 0.7 (0.338) | 0.83 (0.299) | 0.14 (0.098) | 0.76 (0.388) | 0.28 (0.243) | 0.15 (0.098) |
| AABb | 0.6 (0.364) | 0.4 (0.182) | 0.7 (0.384) | 0.5 (0.205) | 0.9 (0.414) | 0.7 (0.241) |
| AAbb | 8 (0.640) | 5 (0.300) | 5 (0.625) | 3 (0.281) | 3 (0.645) | 1.2 (0.194) |
| AaBB | 0.6 (0.364) | 0.4 (0.182) | 0.7 (0.384) | 0.5 (0.205) | 0.9 (0.414) | 0.7 (0.241) |
| AaBb | 5 (0.377) | 10 (0.566) | 3 (0.400) | 5 (0.500) | 1.2 (0.286) | 3 (0.536) |
| Aabb | 0.6 (0.364) | 0.4 (0.182) | 0.7 (0.384) | 0.5 (0.205) | 0.9 (0.414) | 0.7 (0.241) |
| aaBB | 8 (0.640) | 5 (0.300) | 5 (0.625) | 3 (0.281) | 3 (0.645) | 1.2 (0.194) |
| aaBb | 0.6 (0.364) | 0.4 (0.182) | 0.7 (0.384) | 0.5 (0.205) | 0.9 (0.414) | 0.7 (0.241) |
| aabb | 0.6 (0.364) | 0.4 (0.182) | 0.7 (0.384) | 0.5 (0.205) | 0.9 (0.414) | 0.7 (0.241) |

| Pattern 3 | Model 31 | | Model 32 | | Model 33 | |
|---|---|---|---|---|---|---|
| Genotype | $OR_{21}$ ($p_{2\|i}$) | $OR_{31}$ ($p_{3\|i}$) | $OR_{21}$ ($p_{2\|i}$) | $OR_{31}$ ($p_{3\|i}$) | $OR_{21}$ ($p_{2\|i}$) | $OR_{31}$ ($p_{3\|i}$) |
| AABB | 3.27 (0.659) | 1.25 (0.189) | 2.28 (0.543) | 1.56 (0.278) | 1.15 (0.441) | 0.94 (0.271) |
| AABb | 1.5 (0.250) | 5 (0.625) | 1.2 (0.286) | 3 (0.536) | 1.2 (0.348) | 2 (0.435) |
| AAbb | 0.2 (0.170) | 0.3 (0.191) | 0.2 (0.170) | 0.3 (0.191) | 0.2 (0.170) | 0.3 (0.191) |
| AaBB | 4 (0.640) | 2 (0.240) | 3 (0.645) | 1.2 (0.194) | 2 (0.548) | 1.2 (0.247) |
| AaBb | 0.1 (0.108) | 0.1 (0.081) | 0.2 (0.182) | 0.2 (0.136) | 0.5 (0.308) | 0.5 (0.231) |
| Aabb | 1.5 (0.250) | 5 (0.625) | 1.2 (0.286) | 3 (0.536) | 1.2 (0.348) | 2 (0.435) |
| aaBB | 0.3 (0.250) | 0.2 (0.125) | 0.5 (0.323) | 0.3 (0.194) | 0.9 (0.414) | 0.7 (0.241) |
| aaBb | 1.5 (0.250) | 1.5 (0.625) | 1.2 (0.286) | 3 (0.536) | 1.2 (0.348) | 2 (0.435) |
| aabb | 0.3 (0.250) | 0.2 (0.125) | 0.5 (0.323) | 0.3 (0.194) | 0.9 (0.414) | 0.7 (0.241) |

| Pattern 4 | Model 41 | | Model 42 | | Model 43 | |
|---|---|---|---|---|---|---|
| Genotype | $OR_{21}$ ($p_{2\|i}$) | $OR_{31}$ ($p_{3\|i}$) | $OR_{21}$ ($p_{2\|i}$) | $OR_{31}$ ($p_{3\|i}$) | $OR_{21}$ ($p_{2\|i}$) | $OR_{31}$ ($p_{3\|i}$) |
| AABB | 0.67 (0.319) | 0.91 (0.325) | 0.54 (0.320) | 0.54 (0.240) | 0.77 (0.375) | 0.7 (0.258) |
| AABb | 0.6 (0.348) | 0.5 (0.217) | 0.8 (0.372) | 0.8 (0.279) | 0.9 (0.387) | 0.9 (0.290) |
| AAbb | 6 (0.615) | 4 (0.308) | 3 (0.571) | 2 (0.286) | 1.5 (0.476) | 1.2 (0.286) |
| AaBB | 0.5 (0.308) | 0.5 (0.231) | 0.8 (0.372) | 0.8 (0.279) | 0.9 (0.387) | 0.9 (0.290) |
| AaBb | 6 (0.615) | 4 (0.308) | 3 (0.571) | 2 (0.286) | 1.5 (0.476) | 1.2 (0.286) |
| Aabb | 3 (0.377) | 8 (0.615) | 1.2 (0.211) | 5 (0.658) | 1.2 (0.286) | 3 (0.536) |
| aaBB | 6 (0.615) | 4 (0.308) | 3 (0.571) | 2 (0.286) | 1.5 (0.476) | 1.2 (0.286) |
| aaBb | 3 (0.377) | 8 (0.615) | 1.2 (0.211) | 5 (0.658) | 1.2 (0.286) | 3 (0.536) |
| aabb | 0.5 (0.308) | 0.5 (0.231) | 0.8 (0.372) | 0.8 (0.279) | 0.9 (0.387) | 0.9 (0.290) |

| Pattern 5 | Model 51 | | Model 52 | | Model 53 | |
|---|---|---|---|---|---|---|
| Genotype | $OR_{21}$ ($p_{2\|i}$) | $OR_{31}$ ($p_{3\|i}$) | $OR_{21}$ ($p_{2\|i}$) | $OR_{31}$ ($p_{3\|i}$) | $OR_{21}$ ($p_{2\|i}$) | $OR_{31}$ ($p_{3\|i}$) |
| AABB | 0.01 (0.009) | 0.15 (0.128) | 0.11 (0.101) | 0.26 (0.184) | 0.46 (0.280) | 0.58 (0.265) |
| AABb | 6 (0.615) | 4 (0.308) | 3 (0.571) | 2 (0.286) | 1.5 (0.476) | 1.2 (0.286) |
| AAbb | 0.6 (0.364) | 0.4 (0.182) | 0.9 (0.414) | 0.7 (0.241) | 0.9 (0.400) | 0.8 (0.267) |
| AaBB | 6 (0.615) | 4 (0.308) | 3 (0.571) | 2 (0.286) | 1.5 (0.476) | 1.2 (0.286) |
| AaBb | 4 (0.432) | 6 (0.486) | 2 (0.400) | 3 (0.450) | 1.2 (0.390) | 1.5 (0.366) |
| Aabb | 4 (0.432) | 6 (0.486) | 2 (0.400) | 3 (0.450) | 1.2 (0.390) | 1.5 (0.366) |
| aaBB | 0.6 (0.364) | 0.4 (0.182) | 0.9 (0.414) | 0.7 (0.241) | 0.9 (0.400) | 0.8 (0.267) |
| aaBb | 4 (0.432) | 6 (0.486) | 2 (0.400) | 3 (0.450) | 1.2 (0.390) | 1.5 (0.366) |
| aabb | 4 (0.432) | 6 (0.486) | 2 (0.400) | 3 (0.450) | 1.2 (0.390) | 1.5 (0.366) |
