# Supplementary figures and images for: Identification of multiple gene-gene interactions for ordinal phenotypes
Source: BMC Med Genomics. 2013 May 7;6(Suppl 2):S9. doi: 10.1186/1755-8794-6-S2-S9 (PMC3654913; doi:10.1186/1755-8794-6-S2-S9)

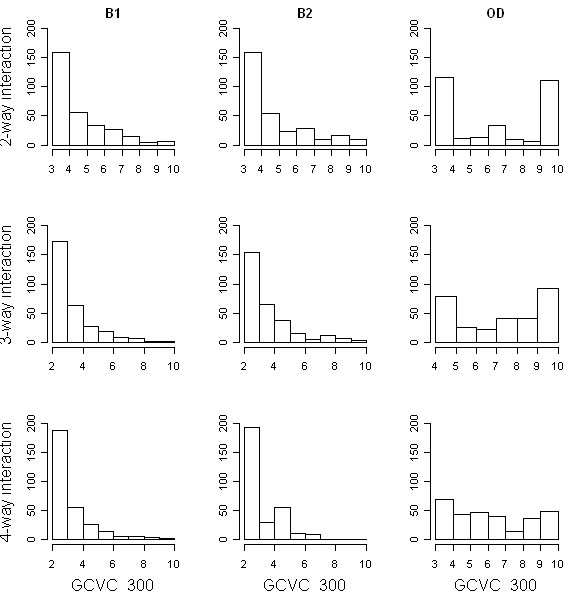

Supplement: Additional file 2 — GCVC value distribution of top-300 OMDR classifiers for 2~4-way interactions from real data analysis. [file 1755-8794-6-S2-S9-S2.tif]
